# Blue Light Increases Neuronal Activity-Regulated Gene Expression in the Absence of Optogenetic Proteins

**DOI:** 10.1523/ENEURO.0085-19.2019

**Published:** 2019-09-17

**Authors:** Kelsey M. Tyssowski, Jesse M. Gray

**Affiliations:** Department of Genetics, Harvard Medical School, Boston, Massachusetts 02115

**Keywords:** activity-regulated genes, immediate early genes, optogenetics, transcription

## Abstract

Optogenetics is widely used to control diverse cellular functions with light, requiring experimenters to expose cells to bright light. Because extended exposure to visible light can be toxic to cells, it is important to characterize the effects of light stimulation on cellular function in the absence of optogenetic proteins. Here we exposed mouse cortical cultures with no exogenous optogenetic proteins to several hours of flashing blue, red, or green light. We found that exposing these cultures to as short as 1 h of blue light, but not red or green light, results in an increase in the expression of neuronal activity-regulated genes. Our findings suggest that blue light stimulation is ill suited to long-term optogenetic experiments, especially those that measure transcription, and they emphasize the importance of performing light-only control experiments in samples without optogenetic proteins.

## Significance Statement

Optogenetics is widely used to control cellular functions using light. For instance, channelrhodopsins, exogenous light-sensitive channels, allow light-dependent control of neuronal firing. This optogenetic control of firing requires exposing neurons to high-powered light. We ask how this light exposure, in the absence of channelrhodopsin, affects the expression of neuronal activity-regulated genes (i.e., the genes that are transcribed in response to neuronal stimuli). Surprisingly, we find that neurons without channelrhodopsin express neuronal activity-regulated genes in response to blue light, but not red or green light, exposure. These findings suggest that experimenters wishing to achieve longer-term (≥1 h) optogenetic control over neuronal firing should avoid using systems that require blue light and should include controls to gauge the effects of light alone.

## Introduction

With the development of optogenetic technologies over the past decade (Boyden et al., 2005; Beyer et al., 2015), it has become increasingly common to expose biological samples to high-powered light. Optogenetics enables light-based control over diverse cellular functions—including neuronal firing (Lin, 2011), transcription (Nihongaki et al., 2015; Polstein and Gersbach, 2015), and cell signaling (Beyer et al., 2015)—via exogenous proteins that are activated by specific wavelengths of light. Results of such experiments can be difficult to interpret when light by itself, in the absence of optogenetic proteins, affects cellular processes. Therefore, it is important to characterize how light exposure affects biological samples.

Light exposure, especially sustained short-wavelength light exposure, can affect cell viability and other cellular processes, including transcription. In cell cultures, including neuronal cultures, hours-long blue or ultraviolet light exposure lowers cell viability via toxic oxidation and free radical formation in the media (Richardson, 1893; Blum, 1932; Stoien and Wang, 1974; Dixit and Cyr, 2003; Wäldchen et al., 2015; Stockley et al., 2017). Light-induced oxidative stress also triggers a transcriptional anti-inflammatory and antioxidative stress response in cultured monocytes (Trotter et al., 2017). Consistent with this idea, cultured microglia exposed to sustained flashing blue light increase the expression of anti-inflammatory genes (Cheng et al., 2016). In neuronal cultures, millisecond-long ultraviolet light exposure increases NMDA currents, and this increase has also been suggested, though not demonstrated, to be caused by oxidative stress (Leszkiewicz et al., 2000). Light can also affect cellular processes *in vivo*. *Drosophila melanogaster* larvae, *Caenorhabditis elegans*, and planaria are sensitive to free radicals that accumulate internally when the animals are exposed to visible light (Bhatla and Horvitz, 2015; Guntur et al., 2015; Birkholz and Beane, 2017), and extended visible light exposure reduces the *C. elegans* life span (De Magalhaes Filho et al., 2018). In addition, briefly exposing the mouse brain to white light triggers GABA release (Wade et al., 1988). Thus, light affects various cellular processes in many cell types, including neurons, both *in vivo* and *in vitro*.

Here we sought to characterize the effects of hours-long light exposure on neural transcription, which could be relevant to studies both within and outside of neuroscience. We were particularly interested in characterizing the effects of light on transcription in neurons because optogenetically driven neuronal activity increases the expression of activity-regulated genes, such as *Fos* (Schoenenberger et al., 2009). Therefore, optogenetics could be a useful tool to precisely control neuronal activation for minutes to hours to study the resulting activity-regulated gene expression. Furthermore, several neuroscience studies on other topics have already used blue light stimulation with exogenous channelrhodopsins to control neuronal firing for hours to days (Goold and Nicoll, 2010; Grubb and Burrone, 2010; Fong et al., 2015; Park et al., 2015). Finally, optogenetics can be used to directly control transcription (Nihongaki et al., 2015; Polstein and Gersbach, 2015) and to control signaling pathways that regulate transcription (Beyer et al., 2015) in both neural and non-neural systems. Therefore, to properly design and interpret optogenetic studies, it is important to understand the effects of hours-long light exposure on gene expression.

We therefore tested whether neuronal activity-regulated gene expression is affected by 1–6 h of blue, red, or green light exposure. We chose light wavelengths that activate published channelrhodopsin variants (Lin, 2011; Lin et al., 2013; Klapoetke et al., 2014) and time points relevant to activity-regulated gene expression (West and Greenberg, 2011; Tyssowski et al., 2018). We found that mixed cortical cultures of neurons and glia that did not express channelrhodopsin showed increased expression of the activity-regulated genes *Fos*, *Npas4*, and *Bdnf* when exposed to 1 or 6 h of blue light, but not when exposed to red or green light. Our findings suggest that light by itself, in the absence of optogenetic proteins, increases the expression of activity-regulated genes. Therefore, experimenters that measure transcription following long-term optogenetic stimulation should take precautions, such as including light-only controls in the absence of optogenetic proteins, to avoid experimental confounds from light-induced increases in gene expression.

## Materials and Methods

### Cell culture

All animal procedures were performed in accordance with the regulations of the Harvard University Animal Care Committee. Cortices were dissected from embryonic day 16 (E16) or postnatal day 0 (P0) to P1 CD1 or C57BL/6 mice of mixed sex. They were dissociated with papain [(L)(S)003126, Worthington]. A total of 150,000–250,000 dissociated cells/well were plated on 48-well Lumos OptiClear plates (Axion), which have opaque well walls and had been coated overnight with poly-ornithine (30 mg/ml; Sigma-Aldrich) and laminin (5 μg/ml) in water and then washed once with PBS. Cultures were maintained at 37^°^C at 5% CO_2_ in BrainPhys media (STEMCELL Technologies) without phenol red supplemented with SM1 (STEMCELL Technologies) and penicillin/streptomycin (Thermo Fisher Scientific). Neurons were used 10–14 d after plating. Replicates performed with E16 and P1 neurons were similar, and therefore were combined in plots and statistical analysis.

### Light stimulation

Light stimulation was performed using the Lumos system programmed with AxIS software with power set at 100% or 50% (Axion Biosystems). According to the manufacturer, 100% power corresponds to 3.9 mW/mm^2^ for blue (475 nm) light, 1.9 mW/mm^2^ for green (530 nm) light, and 2.2 mW/mm^2^ for red (620 nm) light; and 50% power corresponds to 1.95 mW/mm^2^ for blue light. These irradiance measurements were taken from the bottom of a well with no media (Axion Biosystems, personal communication). The temperature was maintained at 36–37^°^C by putting the plate on a 37^°^C warming plate (Bel-Art). The CO_2_ was maintained at 5% throughout the duration of the recording using the base provided with the Axion Lumos system. Neurons were silenced with APV (100 μM; Tocris) and NBQX (10 μM; Tocris) at least 8 h before stimulation to replicate conditions that would be used in optogenetic experiments. Light-exposed wells and wells left in the dark were on the same plate. For E16 experiments, technical replicates were performed from the time of plating (i.e., two to three wells were plated for each condition and used in the experiment). Reported values for each biological replicate are an average of technical replicates (which were similar within each biological replicate). For P1 experiments, two to three wells were plated for each condition, but the mRNA collected from each well was pooled at the time of collection in TRIzol (see below).

### Temperature measurement

We measured temperature using a thermocouple (catalog #5TC-TT-K-30-36, Omega) inserted into a well that was exposed to light stimulation. The temperature on a digital thermometer (VWR) attached to the thermocouple was monitored at the indicated time points.

### RNA extraction and quantitative PCR

Immediately following stimulation, samples were collected in TRIzol (Invitrogen), and total RNA was extracted using the RNeasy Mini Kit (QIAGEN) with in-column DNase treatment (QIAGEN) according to the instructions of the manufacturer. The RNA was then converted to cDNA using the High Capacity cDNA Reverse Transcription kit (Applied Biosystems). For quantitative PCR (qPCR), we used SsoFast Evagreen supermix (Bio-Rad) with primers in Table 1 and ran qPCR on a Bio-Rad CFX384 thermocycler using the following cycling conditions: 95^º^C for 3 min, repeat 40× (95^°^C for 5 s, 60^°^C for 15 s), 65^°^C for 5 s, and 95^°^C for 5 s. We performed two technical replicates for each sample in each qPCR experiment and used the average in analysis.

**Table 1:** qPCR primers

Gene	Primer
*Fos* (fw)	GGCTCTCCTGTCAACACACA
*Fos* (rv)	TGTCACCGTGGGGATAAAGT
*Npas4* (fw)	GTTGCATCAACTCCAGAGCCAAGT
*Npas4* (rv)	ACATTTGGGCTGGACCTACCTTCA
*Bdnf* (fw)	TCCACCAGGTGAGAGTG
*Bdnf* (rv)	GCCTTCATGCAACCGAAGTA
*Thy1* (fw)	GAAAACTGCGGGCTTCAG
*Thy1* (rv)	CCAAGAGTTCCGACTTGGAT
*Tubb3* (fw)	CGACAATGAAGCCCTCTACGAC
*Tubb3* (rv)	ATGGTGGCAGACACAAGGTGGTTG
*Gfap* (fw)	TCCTGGAACAGCAAAACAAG
*Gfap* (rv)	CAGCCTCAGGTTGGTTTCAT
*Cx3cr1* (fw)	CAGCATCGACCGGTACCTT
*Cx3cr1* (rv)	GCTGCACTGTCCGGTTGTT
*Gapdh* (fw)	CGTCCCGTAGACAAAATGGT
*Gapdh* (rv)	TCGTTGATGGCAACAATCTC

### Analysis and statistics

For qPCR analysis, we use the method of Pfaffl (2004; Bio-Rad Laboratories, 2006) to calculate relative gene expression values based on Ct values. Specifically, we made a dilution series of cDNA from the same experiment for each primer and used that to make a standard curve that allowed us to determine primer efficiency. We then used that standard curve to convert Ct values into relative expression values for each primer set, as described by Pfaffl (2004). We then normalized our neuronal activity-regulated gene expression values by values for the housekeeping gene *Gapdh* to control for any differences in the amount of cDNA in each reaction. For all conditions, *Gapdh* fold changes were between 0.80 and 1.36 (Table 2). Furthermore, *Gapdh* mRNA is highly expressed and highly stable, making it less likely to be altered by small changes in transcription. Each biological replicate was from a different dissection on a different day. For E16 experiments, different biological replicates were run on separate qPCR plates, and for P1 experiments, different biological replicates were run on the same qPCR plate. The *t* tests testing fold change were performed on log fold change values from biological replicates testing the difference from a fold change of 1. Fold change was calculated for each biological replicate as the *Gapdh*-normalized expression from the stimulated culture divided by the *Gapdh*-normalized expression from an unstimulated culture (i.e., those not exposed to light). The means and SDs of these normalized expression values are shown in Table 3.

**Table 2:** *Gapdh* fold change

Condition	Mean fold change	*p* Value	*q* Value
475 nm, 1 h, 10 Hz, 3.9 mW/mm^2^	0.92	0.45*^jj^*	0.78
475 nm, 6 h, 10 Hz, 3.9 mW/mm^2^	1.25	0.45*^kk^*	0.78
612 nm, 1 h, 10 Hz, 2.2 mW/mm^2^	0.80	0.43*^ll^*	0.78
612 nm, 6 h, 10 Hz, 2.2 mW/mm^2^	1.20	0.76*^mm^*	0.88
530 nm, 1 h, 10 Hz, 1.9 mW/mm^2^	1.21	0.78*^nn^*	0.88
530 nm, 6 h, 10 Hz, 1.9 mW/mm^2^	1.14	0.88*^oo^*	0.88
475 nm, 6 h, 100 Hz, 3.9 mW/mm^2^	0.81	0.26*^pp^*	0.78
475 nm, 6 h, 100 Hz, 1.95 mW/mm^2^	0.86	0.52*^qq^*	0.78
612 nm, 6 h, 100 Hz, 2.2 mW/mm^2^	1.36	0.02*^rr^*	0.19

*p* Values were obtained from a *t* test on log fold change values testing a difference from a fold change of 1. *q* Values were obtained from multiple hypothesis adjustment using FDR for all of the *p* values in this table.

**Table 3:** SDs and means for normalized expression values for each condition

Gene/condition	SD	Mean
*Fos*, no light	0.871	1.06
*Fos*, 475 nm, 10 Hz, 3.9 mW/mm^2^, 1 h	0.782	1.57
*Fos*, 475 nm, 10 Hz, 3.9 mW/mm^2^, 6 h	0.822	2.29
*Fos*, 612 nm, 10 Hz, 2.2 mW/mm^2^, 1 h	0.0523	0.614
*Fos*, 612 nm, 10 Hz, 2.2 mW/mm^2^, 6 h	0.0834	0.749
*Fos*, 530 nm, 10 Hz, 1.9 mW/mm^2^, 1 h	0.0241	0.479
*Fos*, 530 nm, 10 Hz, 1.9 mW/mm^2^, 6 h	0.124	0.614
*Fos*, 475 nm, 100 Hz, 3.9 mW/mm^2^, 6 h	7.08	12
*Fos*, 475 nm, 100 Hz, 1.95 mW/mm^2^, 6 h	2.97	6.69
*Fos*, 612 nm, 100 Hz, 2.2 mW/mm^2^, 6 h	0.452	0.885
*Bdnf*, no light	0.293	0.771
*Bdnf*, 475 nm, 10 Hz, 3.9 mW/mm^2^, 6 h	1.03	2.26
*Bdnf*, 612 nm, 10 Hz, 2.2 mW/mm^2^, 6 h	0.587	1.07
*Bdnf*, 530 nm, 10 Hz, 1.9 mW/mm^2^, 6 h	0.195	0.794
*Npas4*, no light	0.314	0.902
*Npas4*, 475 nm, 10 Hz, 3.9 mW/mm^2^, 6 h	0.784	1.9
*Npas4*, 612 nm, 10 Hz, 2.2 mW/mm^2^, 6 h	0.388	1.02
*Npas4*, 530 nm, 10 Hz, 1.9 mW/mm^2^, 6 h	0.26	0.829
*Thy1*, no light	0.782	1.84
*Thy1*, 475 nm, 100 Hz, 3.9 mW/mm^2^, 6 h	0.812	1.69
*Thy1*, 475 nm, 100 Hz, 1.95 mW/mm^2^, 6 h	2.24	2.9
*Thy1*, 612 nm, 100 Hz, 2.2 mW/mm^2^, 6 h	1.05	2.1
*Tubb3*, no light	0.268	1.06
*Tubb3*, 475 nm, 100 Hz, 3.9 mW/mm^2^, 6 h	0.0973	0.477
*Tubb3*, 475 nm, 100 Hz, 1.95 mW/mm^2^, 6 h	0.0779	0.616
*Tubb3*, 612 nm, 100 Hz, 2.2 mW/mm^2^, 6 h	0.163	0.799
*Gfap*, no light	1.06	2.02
*Gfap*, 475 nm, 100 Hz, 3.9 mW/mm^2^, 6 h	0.366	0.799
*Gfap*, 475 nm, 100 Hz, 1.95 mW/mm^2^, 6 h	0.323	1.12
*Gfap*, 612 nm, 100 Hz, 2.2 mW/mm^2^, 6 h	0.621	1.39
*Cx3cr1*, no light	1.74	3.11
*Cx3cr1*, 475 nm, 100 Hz, 3.9 mW/mm^2^, 6 h	0.0489	0.144
*Cx3cr1*, 475 nm, 100 Hz, 1.95 mW/mm^2^, 6 h	0.0866	0.465
*Cx3cr1*, 612 nm, 100 Hz, 2.2 mW/mm^2^, 6 h	0.368	1.39

**Figure 1.**
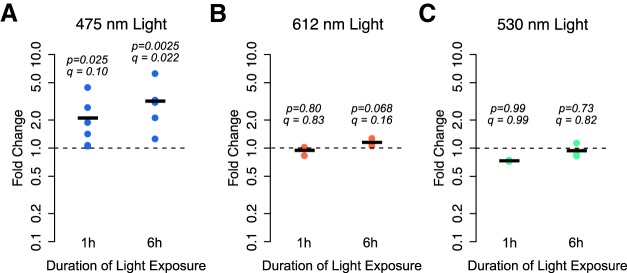
***A–C***, Cortical cultures without exogenous channelrhodopsin were exposed to a pattern of 10 Hz, 2 ms pulses of 475 nm (blue; ***A***), 612 nm (red; ***B***), or 530 nm (green; ***C***) light for 1 or 6 h. The expression of the activity-regulated gene *Fos* was measured using quantitative real-time PCR. Values plotted are the fold change in mRNA expression at 1 or 6 h compared with cortical cultures not exposed to light. Black lines represent the average of *n* = 3-6 biological replicates (each from a different cortical dissection), and dots are the values from each replicate. *p* Values are from a one-sided Student’s *t* test on log fold changes testing an increase from a fold change of 1 (no change). *q* Values are from FDR adjustment of all *p* values in this article that test the hypotheses that gene expression increases or changes in response to light exposure.

**Figure 2.**
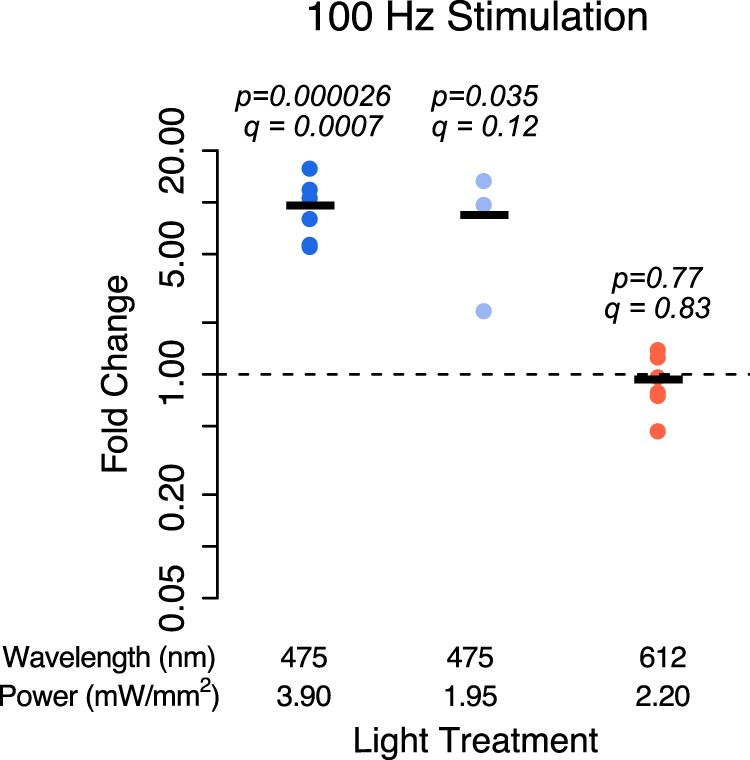
Cortical cultures without exogenous channelrhodopsin were exposed to a pattern of 100 Hz, 1 ms pulses of 475 nm (blue) or 612 nm (red) light for 6 h. Blue light was used at two light powers, 3.9 and 1.95 mW/mm^2^; and red light was used at 2.2 mW/mm^2^. Expression of the activity-regulated gene *Fos* was measured using quantitative real-time PCR. The values plotted are the fold change in mRNA expression after 6 h of light stimulation compared with cultures not exposed to light. Black lines represent the average of *n* = 3-6 biological replicates (each from different a cortical dissection), and dots are the values from each replicate. *p* Values are from a one-sided Student’s *t* test on log fold changes testing an increase from a fold change of 1 (no change). *q* Values are from FDR adjustment of all *p* values in this article that test the hypotheses that gene expression increases or changes in response to light exposure.

We performed statistics using R. We performed one-sided *t* tests on data testing the hypothesis that activity-regulated gene expression increases with light stimulation, as we wished to focus on the increase. We used two-sided *t* tests for all other comparisons. Information on statistical tests is in Table 4. We adjusted all of our *p* values for multiple hypothesis testing by using the Benjamini–Hochberg false discovery rate (FDR) correction in the R function p.adjust to generate *q* values. We performed FDR on *p* values from all experiments that tested the hypotheses that “gene expression increases or changes with light stimulation” (Figs. 1, 2; see Figs. 4, 5). We used a *q* value threshold of 0.15 to call “significance.” To address the statistical likelihood that we would observe under multiple experimental conditions (e.g., time points) that blue light, but not red or green light, increases activity-regulated gene expression, we performed a bootstrapping analysis. We randomized the data from all experiments that tested activity-regulated gene expression. Specifically, we permuted observed fold change values from each replicate across all replicates and conditions such that each condition was assigned a number of fold change values equal to the number of replicates in our actual experiments. We then determined a *p* value for each permuted condition. In 10,000 repetitions, we never observed the results that we observed in our actual experiments: a significant change (*p* < 0.05) for blue light-treated samples, but not red light- or green light-treated samples. These results indicate that the results we observed are unlikely by chance (*p* < 0.0001).

**Table 4:** Statistical table

	Data structure	Type of test (log = natural log)	95% CI (lower bound for one-sided tests)	Experiment	Figures/tables
*a*	Normal	One-sided Student’s *t* test on log fold change, test difference from log(1)	0.1319194	Fos 1 h, blue, 10 Hz	Figure 1
*b*	Normal	One-sided Student’s *t* test on log fold change, test difference from log(1)	0.6031834	Fos 6 h, blue, 10 Hz	Figure 1
*c*	Normal	One-sided Student’s *t* test on log fold change, test difference from log(1)	−0.249898	Fos 1 h, red, 10 Hz	Figure 1
*d*	Normal	One-sided Student’s *t* test on log fold change, test difference from log(1)	−0.02737114	Fos 6 h, red, 10 Hz	Figure 1
*e*	Normal	One-sided Student’s *t* test on log fold change, test difference from log(1)	−0.8537	Fos 1 h, green, 10 Hz	Figure 1
*f*	Normal	One-sided Student’s *t* test on log fold change, test difference from log(1)	−0.3404513	Fos 6 h, green, 10 Hz	Figure 1
*g*	Normal	One-sided Student’s *t* test on log fold change, test difference from log(1)	1.83808	Fos, 6 h, blue, 100 Hz	Figure 2
*h*	Normal	One-sided Student’s *t* test on log fold change, test difference from log(1)	0.3375841	Fos, 6 h, blue, low power, 100 Hz	Figure 2
*i*	Normal	One-sided Student’s *t* test on log fold change, test difference from log(1)	−0.456429	Fos, 6 h, red, 100 Hz	Figure 2
*j*	Normal	Two-sided Student’s *t* test on log fold changes	0.521899 1.763154	Fos 100 Hz vs 10 Hz, blue	Figures 1, 2
*k*	Normal	Two-sided Student’s *t* test on log fold changes	−1.774112 2.339614	Fos 100 Hz high power vs Fos 100 Hz low power	Figure 2
*l*	Normal	Two-sided Student’s *t* test	−1.5504421 −0.1162245	Temperature: 10 Hz blue vs 100 Hz blue at 5 min	Figure 3
*m*	Normal	Two-sided Student’s *t* test	−3.926551 2.426551	Temperature: 100 Hz blue vs 100 Hz red at 5 min	Figure 3
*n*	Normal	Two-sided Student’s *t* test	−1.38377545 0.05044212	Temperature: 10 Hz blue vs 100 Hz blue at 15 min	Figure 3
*o*	Normal	Two-sided Student’s *t* test	−1.7777220 0.9443886	Temperature: 100 Hz blue vs 100 Hz red at 15 min	Figure 3
*p*	Normal	Two-sided Student’s *t* test	−1.5504421 −0.1162245	Temperature: 10 Hz blue vs 100 Hz blue at 6 h	Figure 3
*q*	Normal	Two-sided Student’s *t* test	−3.426551 2.926551	Temperature: 100 Hz blue vs 100 Hz red at 6 h	Figure 3
*r*	Normal	One-sided Student’s *t* test on log fold change, test difference from log(1)	0.144	Bdnf, 6 h, blue, 10 Hz	Figure 4
*s*	Normal	One-sided Student’s *t* test on log fold change, test difference from log(1)	−0.061	Bdnf, 6 h, red, 10 Hz	Figure 4
*t*	Normal	One-sided Student’s *t* test on log fold change, test difference from log(1)	−0.52	Bdnf, 6 h, green, 10 Hz	Figure 4
*u*	Normal	One-sided Student’s *t* test on log fold change, test difference from log(1)	0.2876212	Npas4, 6 h, blue, 10 Hz	Figure 4
*v*	Normal	One-sided Student’s *t* test on log fold change, test difference from log(1)	−0.05463496	Npas4, 6 h, red, 10 Hz	Figure 4
*w*	Normal	One-sided Student’s *t* test on log fold change, test difference from log(1)	−0.6481334	Npas4, 6 h, green, 10 Hz	Figure 4
*x*	Normal	Two-sided Student’s *t* test on log fold changes, testing difference from log(1)	−0.23030841 0.02717987	Thy1, 6 h, blue, 100 Hz, 3.9 mW/mm^2^	Figure 4
*y*	Normal	Two-sided Student’s *t* test on log fold changes, testing difference from log(1)	−0.4856258 1.1311706	Thy1, 6 h, blue, 100 Hz, 2.95 mW/mm^2^	Figure 4
*z*	Normal	Two-sided Student’s *t* test on log fold changes, testing difference from log(1)	−0.1336059 0.3427545	Thy1, 6 h, red, 100 Hz	Figure 4
*aa*	Normal	Two-sided Student’s *t* test on log fold changes, testing difference from log(1)	−1.9422738 0.3562803	Tubb3, 6 h, blue, 100 Hz, 3.9 mW/mm^2^	Figure 4
*bb*	Normal	Two-sided Student’s *t* test on log fold changes, testing difference from log(1)	−1.14604760 0.08778751	Tubb3, 6 h, blue, 100 Hz, 2.95 mW/mm^2^	Figure 4
*cc*	Normal	Two-sided Student’s *t* test on log fold changes, testing difference from log(1)	−0.7474299 0.1929174	Tubb3, 6 h, red, 100 Hz	Figure 4
*dd*	Normal	Two-sided Student’s *t* test on log fold changes, testing difference from log(1)	−1.2076411 −0.5625319	Gfap, 6 h, blue, 100 Hz, 3.9 mW/mm^2^	Figure 5
*ee*	Normal	Two-sided Student’s *t* test on log fold changes, testing difference from log(1)	−1.6217416 0.6494064	Gfap, 6 h, blue, 100 Hz, 2.95 mW/mm^2^	Figure 5
*ff*	Normal	Two-sided Student’s *t* test on log fold changes, testing difference from log(1)	−1.1985427 0.5754028	Gfap, 6 h, red, 100 Hz	Figure 5
*gg*	Normal	Two-sided Student’s *t* test on log fold changes, testing difference from log(1)	−3.635956 −2.350329	Cx3r1, 6 h, blue, 100 Hz, 3.9 mW/mm^2^	Figure 5
*hh*	Normal	Two-sided Student’s *t* test on log fold changes, testing difference from log(1)	−3.53367205 −0.05504915	Cx3cr1, 6 h, blue, 100 Hz, 2.95 mW/mm^2^	Figure 5
*ii*	Normal	Two-sided Student’s *t* test on log fold changes, testing difference from log(1)	−1.6631094 0.2506975	Cx3cr1, 6 h, red, 100 Hz	Figure 5
*jj*	Normal	Two-sided Student’s *t* test on log fold changes, testing difference from log(1)	−0.729 0.379	Gapdh, 1 h, blue, 10 Hz, 3.9 mW/mm^2^	Table 2
*kk*	Normal	Two-sided Student’s *t* test on log fold changes, testing difference from log(1)	−0.319 0.621	Gapdh, 6 h, blue, 10 Hz, 3.9 mW/mm^2^	Table 2
*ll*	Normal	Two-sided Student’s *t* test on log fold changes, testing difference from log(1)	−1.778 1.118	Gapdh, 1 h, red, 10 Hz, 2.2 mW/mm^2^	Table 2
*mm*	Normal	Two-sided Student’s *t* test on log fold changes, testing difference from log(1)	−1.144 1.345	Gapdh, 6 h, red, 10 Hz, 2.2 mW/mm^2^	Table 2
*nn*	Normal	Two-sided Student’s *t* test on log fold changes, testing difference from log(1)	−1.259 1.462	Gapdh, 1 h, green, 10 Hz, 1.9 mW/mm^2^	Table 2
*oo*	Normal	Two-sided Student’s *t* test on log fold changes, testing difference from log(1)	−2.320 2.144	Gapdh, 6 h, green, 10 Hz, 1.9 mW/mm^2^	Table 2
*pp*	Normal	Two-sided Student’s *t* test on log fold changes, testing difference from log(1)	−1.303 0.435	Gapdh, 6 h, blue, 100 Hz, 3.9 mW/mm^2^	Table 2
*qq*	Normal	Two-sided Student’s *t* test on log fold changes, testing difference from log(1)	−1.568 1.087	Gapdh, 6 h, blue, 10 Hz, 1.95 mW/mm^2^	Table 2
*rr*	Normal	Two-sided Student’s *t* test on log fold changes, testing difference from log(1)	0.063 0.510	Gapdh, 6 h, red, 10 Hz, 2.2 mW/mm^2^	Table 2

### Neuronal activity measurement

Neuronal activity was measured using neurons plated on Lumos Axion MEA plates coated as described above. Lumos MEA plates have 48 wells, each containing 16 PEDOT [poly(3,4-ethylenedioxythiophene)] electrodes in a 4 × 4 grid. Electrodes are 50 μm in diameter and spaced 350 μm apart. Neurons from P0 or P1 mice were dissociated and cultured as described above. Recordings were made using Maestro and MiddleMan from Axion Biosystems (version 1.0.0.0), along with AxIS software (version 2.4.5). Neurons were kept at 37^°^C with 5% CO_2_ during recordings using the Axion Maestro system. Raw data were filtered in AxIS on-line using a 200 Hz Butterworth high-pass filter and a 3000 Hz Butterworth low-pass filter. Spikes were detected in AxIS on-line using peak detection with an adaptive threshold of 5.5 SDs from noise levels. To avoid the detection of overlapping spikes, detection was prevented for 2.16 ms after each peak.

## Results

To determine the effect of light exposure on cortical cultures, we exposed mixed mouse cortical cultures of neurons and glia that did not express exogenous channelrhodopsin to a pattern of blue light (475 nm) consisting of 2 ms pulses at a frequency of 10 Hz. We used a light intensity of 3.9 mW/mm^2^ (see Materials and Methods), which is similar to, or less than, the light intensity recommended for optogenetic activation of channelrhodopsin and similar molecules (Lin, 2011; Lin et al., 2013; Klapoetke et al., 2014). After light exposure, we assessed the mRNA expression of the neuronal activity-regulated gene *Fos* using qPCR. We found that cultures exposed to just 1 h of 10 Hz flashing blue light had 2.1-fold higher *Fos* mRNA expression than cultures left in the dark (Fig. 1*A*; *p* = 0.025*^a^*, *q* = 0.10). Following 6 h of light exposure, we observed a 3.2-fold increase in *Fos* mRNA expression compared to cultures left in the dark (*p* = 0.0025*^b^*, *q* = 0.022), suggesting that blue light exposure—in the absence of optogenetic proteins—increases *Fos* mRNA expression.

We next asked whether *Fos* mRNA expression is increased by exposure to red light (612 nm) or green light (530 nm). We exposed cortical cultures to 6 h of the same 10 Hz pattern and found that neither red nor green light exposure increased *Fos* expression >1.2-fold (Fig. 1*B*,*C*; 1 h red light exposure: fold change = 1.1, *p* = 0.80*^c^*, *q* = 0.83; 6 h red light exposure: fold change = 0.94, *p* = 0.068*^d^*, *q* = 0.16; 1 h green light exposure: fold change = 0.73, *p* = 0.99*^e^*, *q* = 0.99; 6 h green light exposure: fold change = 0.94, *p* = 0.73*^f^*, *q* = 0.82).

We then asked whether increasing the frequency of blue light exposure results in even higher mRNA expression. When we changed the pattern of blue light stimulation to a frequency of 100 Hz, we found that cultures showed a 9.5-fold increase in *Fos* expression after 6 h of light exposure (Fig. 2; *p* = 0.000026 × *g*, *q* = 0.0007). This was more than the 3.1-fold increase we saw after 6 h of exposure to 10 Hz blue light (*p* = 0.002*^j^*, *t* test), indicating that more light exposure results in a greater increase in *Fos* mRNA expression (Fig. 2). However, we found that for red light, even the 100 Hz stimulation pattern failed to increase *Fos* expression (*p* = 0.77*^i^*, *q* = 0.83, fold change = 0.93).

The failure of red light to increase gene expression could be due to the fact that we used a lower power for red light stimulation (2.2 mW/mm^2^) than for blue light stimulation (3.9 mW/mm^2^), or it could indicate that *Fos* is particularly sensitive to blue light. To distinguish between these possibilities, we stimulated cultures in the same 100 Hz pattern with lower-power (1.95 mW/mm^2^) blue light. We found that cultures stimulated with lower-power light still exhibited 8.4-fold higher *Fos* expression compared with unstimulated controls (*p* = 0.035*^h^*, *q* = 0.12), similar to the fold change we observed with higher-power light (*p* = 0.66*^k^*, *t* test). The finding that blue light increases *Fos* expression whereas red light at a similar power does not indicates that the light-driven increase in *Fos* expression is specific to short-wavelength light exposure.

**Figure 3.**
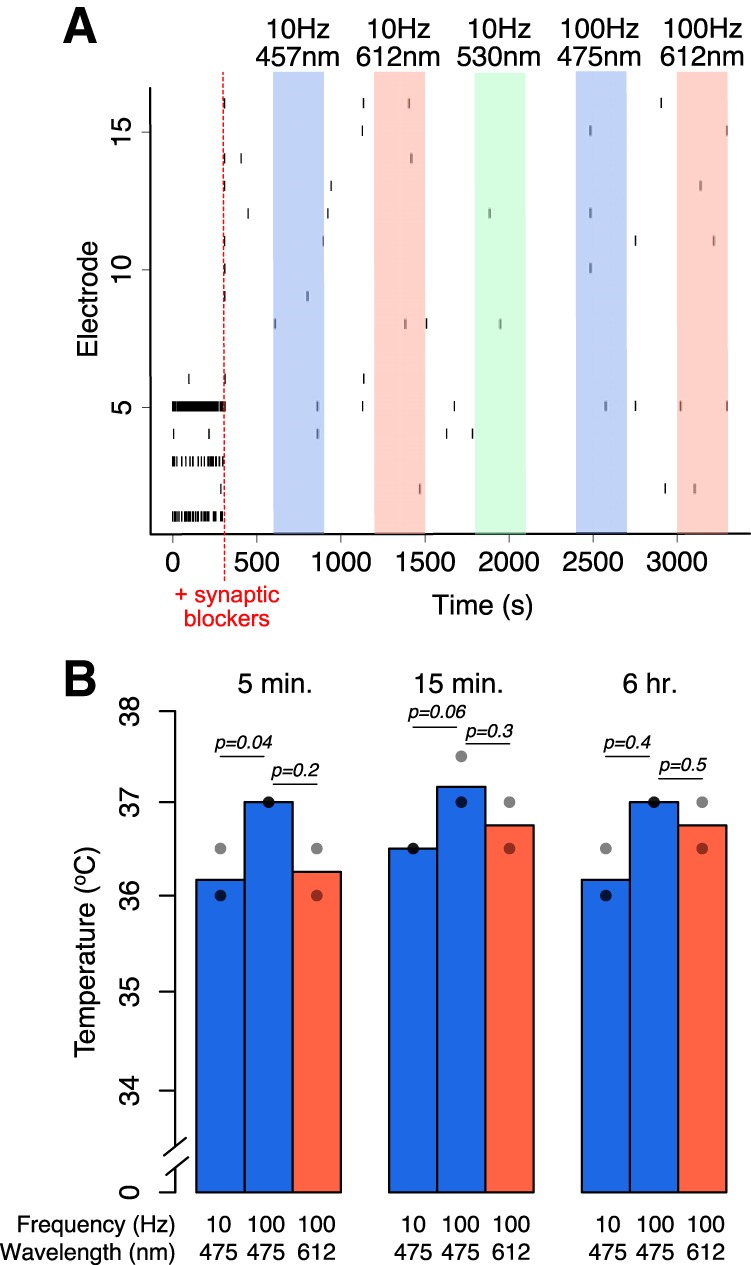
***A***, Cortical cultures without exogenous channelrhodopsin plated on multielectrode arrays were exposed to the indicated light conditions. As in all experiments, neurons were silenced before light exposure with synaptic blockers APV and NBQX. Each line represents an action potential. Red, green, or blue light is ON at the highlighted times. Representative example from one experiment. ***B***, Temperature measurements were taken from a well exposed to blue light at several time points during the course of a 6 h experiment. All wells began at 36^°^C after an adjustment period of at least 1 h on the warming plate and Axion Lumos system. Results from *n* = 2-3 replicates performed on different days. *p* Values are from a two-sided Student’s *t* test.

We next investigated whether increased *Fos* expression might be due to one of several possible secondary effects of blue light exposure. Neuronal *Fos* expression increases as a result of the membrane depolarization that occurs during an action potential. However, we did not observe an increase in action potential firing when neurons without channelrhodopsin grown on multielectrode arrays were exposed to light for short periods (Fig. 3*A*), suggesting that blue light-induced membrane depolarization is not the cause of the observed increase in *Fos* expression. We cannot, however, rule out the possibility that longer exposure to blue light stimulation may increase neuronal activity. We also confirmed that the sustained blue light exposure did not substantially alter the temperature of the media. While 100 Hz stimulation initially increased the temperature compared with 10 Hz stimulation by ∼1^°^C (5 min, *p* = 0.04*^l^*; 15 min, *p* = 0.06*^n^*; 6 h, *p* = 0.4*^p^*), even with 100 Hz stimulation, the media remained between 36 and 37.5^°^C for the duration of the experiment (Fig. 3*B*). Although small changes in temperature may affect cellular processes (Ait Ouares et al., 2019; Owen et al., 2019), cultures exposed to 100 Hz red light, which does not increase *Fos* expression, exhibited increases in media temperature similar to those of 100 Hz blue light treatment (5 min, *p* = 0.2*^m^*; 15 min, *p* = 0.3*^°^*; 6 h, *p* = 0.5*^q^*), suggesting that an increase in temperature is unlikely to drive increased gene expression.

**Figure 4.**
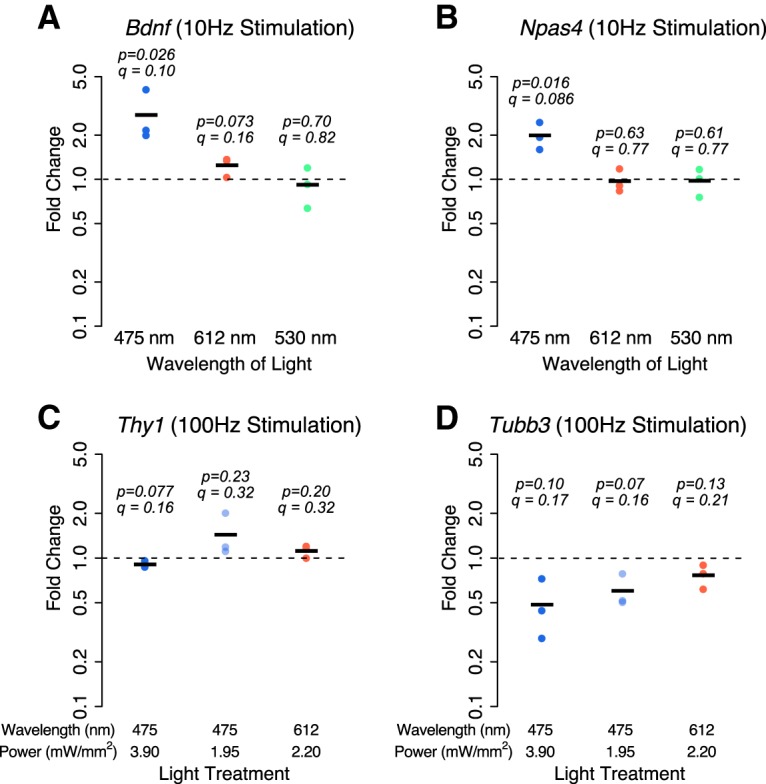
***A***, ***B***, Cortical cultures without exogenous channelrhodopsin were exposed to a pattern of 10 Hz, 2 ms pulses of 475 nm (blue), 612 nm (red), or 530 nm (green) light for 6 h (A and B); or 100 Hz, 1 ms pulses of 3.9 mW/mm^2^ (475 nm), 1.95 nW/mm^2^ (475 nm), or 2.2 nW/mm^2^ (612 nm) light for 6 h. ***A–D***, Expression of the activity-regulated genes *Bdnf* (***A***) and *Npas4* (***B***), or the neuronal marker genes *Thy1* (***C***) and *Tubb3* (***D***), was measured using quantitative real-time PCR. Values plotted are the fold change in mRNA expression at 6 h compared with cultures not exposed to light. Black lines represent the average of *n* = 3 biological replicates (from separate cortical dissections), and dots are the values from each replicate. The *p* values are from a one-sided (***A***, ***B***) or two-sided (***C***, ***D***) Student’s *t* test on log fold changes testing an increase (***A***, ***B***) or a change (***C***, ***D***) from a fold change of 1 (no change). *q* Values are from FDR adjustment of all *p* values in this article that test the hypotheses that gene expression increases or changes in response to light exposure.

Next, we asked whether light exposure increases the expression of others of the hundreds of neuronal activity-regulated genes. Specifically, we assessed the expression of *Bdnf* and *Npas4* mRNA using qPCR. We hypothesized that since *Bdnf* is regulated differently from *Fos* (West and Greenberg, 2011; Tyssowski et al., 2018), it may not be affected by the *Fos*-regulating signaling pathways activated by blue light exposure. However, we found that *Bdnf* mRNA expression is increased 2.7-fold by a 6 h exposure to blue light (*p* = 0.026*^r^*, *q* = 0.10), but not red light (fold change = 1.2, *p* = 0.073*^s^*, *q* = 0.16) or green light (fold change = 0.92, *p* = 0.70*^t^*, *q* = 0.82; Fig. 4*A*). Increased expression of *Npas4* mRNA, unlike *Fos*, is relatively specific to activated neurons (Lin et al., 2008; Fowler et al., 2011). We thus reasoned that if the increases in gene expression in response to blue light stimulation were activated as part of a response to oxidation and cell death (Richardson, 1893; Blum, 1932; Stoien and Wang, 1974), a neuronal activation-specific gene might not increase in expression. However, we found that a 6 h exposure to blue light (*p* = 0.016*^u^*, *q* = 0.086), but not red light (fold change = 0.97, *p* = 0.63*^v^*, *q* = 0.77) or green light (fold change = 0.98, *p* = 0.61*^w^*, *q* = 0.77), also resulted in a twofold increase in *Npas4* mRNA expression (Fig. 4*B*). We therefore suspect that many neuronal activity-regulated genes increase their expression in response to blue light exposure. Interestingly, we found that neither the excitatory neuron marker gene *Thy1* (Fig. 4*C*; high-power blue light, *p* = 0.077*^x^*, *q* = 0.16; low-power blue light, *p* = 0.23*^y^*, *q* = 0.32; red light, *p* = 0.20*^z^*, *q* = 0.32) nor the neuronal gene *Tubb3* (Fig. 4*D*; high-power blue light, *p* = 0.10*^aa^*, *q* = 0.17; low-power blue light, *p* = 0.07*^bb^*, *q* = 0.16; red light, *p* = 0.13*^cc^*, *q* = 0.21) showed increased mRNA expression in response to 100 Hz light stimulation, suggesting that the blue light-driven increases in gene expression may be specific to activity-regulated genes.

**Figure 5.**
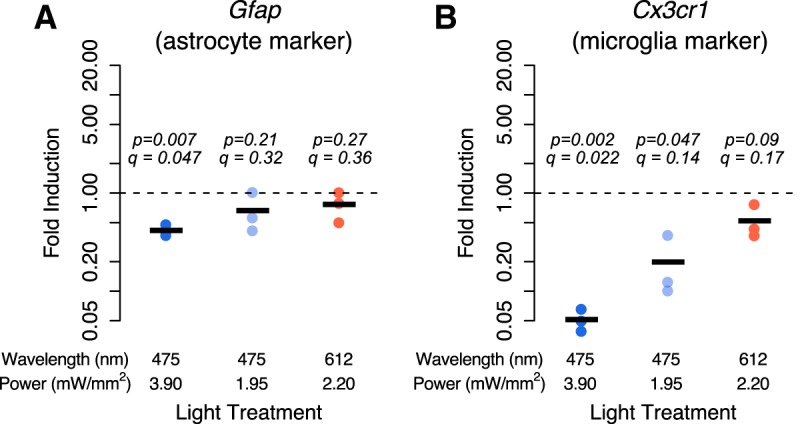
Cortical cultures without channelrhodopsin were exposed to a pattern of 100 Hz, 1 ms pulses of 3.9 mW/mm^2^ (475 nm), 1.95 nW/mm^2^ (475 nm), or 2.2 nW/mm^2^ (612 nm) light for 6 h. ***A***, ***B***, The expression of the astrocyte marker *Gfap* (***A***) and microglial marker *Cx3cr1* (***B***) were measured using quantitative real-time PCR. Values plotted are the fold change in mRNA expression at 6 h compared with cultures not exposed to light. Black lines represent the average of *n* = 3 biological replicates (from separate cortical dissections), and dots are the values from each replicate. *p* Values are from a two-sided Student’s *t* test on log fold changes testing a change from a fold change of 1 (no change). *q* Values are from FDR adjustment of all *p* values in this article that test the hypotheses that gene expression increases or changes in response to light exposure.

Finally, we asked whether light exposure might affect the expression of non-neuronal genes, as our cultures contain other neural cell types. We thus measured the expression of the astrocyte marker gene *Gfap* and the microglia marker gene *Cx3cr1* (Hrvatin et al., 2018) in cultures treated for 6 h with 100 Hz 3.9 mW/mm^2^ blue light, 10 Hz 3.9 mW/mm^2^ blue light, 100 Hz 1.95 mW/mm^2^ blue light, and 100 Hz 2.2 mW/mm^2^ red light. We observed a 2.4-fold decrease in *Gfap* expression in cultures treated with 100 Hz blue light (Fig. 5*A*; *p* = 0.007*^dd^*, *q* = 0.047). We further observed that the expression of the microglial marker gene *Cx3cr1* was dramatically reduced by blue light exposure (up to 20-fold; 3.9 mW/mm^2^: *p* = 0.002*^gg^*, *q* = 0.022; 1.95 mW/mm^2^: *p* = 0.047*^hh^*, *q* = 0.14). We also observed a twofold decrease in *Cx3cr1* in response to red light treatment, albeit with *p* = 0.09*^ii^*, *q* = 0.17 (Fig. 5*B*). These light-induced decreases in marker gene expression could indicate either that marker gene expression is altered by light stimulation, perhaps underlying previously reported changed in morphology (Stockley et al., 2017), or that astrocytes or microglia are killed by light stimulation.

## Discussion

We show in cortical cultures without exogenous channelrhodopsin that extended exposure to blue light resulted in a greater than twofold increase in the expression of neuronal activity-regulated genes. This increase in gene expression does not occur in response to exposure to red or green light. We further find that extended exposure to blue light also decreases the expression of microglia and astrocyte marker genes, which could indicate that extended light exposure kills non-neuronal cells. Our findings suggest that blue light is ill suited to optogenetic experiments that use long-term light exposure and those that assess changes in activity-regulated gene transcription in response to optogenetic stimulation. This work also emphasizes the importance of including experimental controls in optogenetic experiments that allow experimenters to determine the effects of light on cells in the absence of exogenous light-activated proteins (Allen et al., 2015).

Our finding that blue, but not red or green, light increases the expression of neuronal activity-regulated genes is consistent with other work demonstrating detrimental effects of short-wavelength light (Stoien and Wang, 1974; Godley et al., 2005; Cheng et al., 2016; Stockley et al., 2017). Several studies that have compared the effects of blue light to other wavelengths of light both *in vitro* and in *C. elegans* have found that blue light has greater effects on cell viability (Wäldchen et al., 2015), *C. elegans* behavior (Bhatla and Horvitz, 2015), and *C. elegans* survival (De Magalhaes Filho et al., 2018). These data suggest that using optogenetic proteins that are activated by longer wavelengths of light (Lin et al., 2013; Klapoetke et al., 2014) might allow experimenters to avoid side effects of light exposure. However, as we still observe a potential decrease in microglial gene expression in response to red light, using longer wavelength light likely cannot prevent all side effects of light exposure.

We speculate that the expression of activity-regulated genes increases in response to blue light due to the oxidation that occurs in biological liquids in response to extended light exposure (Richardson, 1893; Blum, 1932; Stoien and Wang, 1974; Dixit and Cyr, 2003; Stockley et al., 2017). Oxidative stress can induce the transcription of primary response genes, including *Fos*, in a variety of cell types via activation of cell-signaling pathways, including the MAPK and nuclear factor-κB pathways (Allen and Tresini, 2000). Because oxidative stress activates pathways similar to those of neuronal activity (West and Greenberg, 2011), we might expect oxidative stress to activate many neuronal activity-regulated genes without activating neuronal marker genes. Indeed, we observed that blue light exposure increases the expression of all three of the neuronal activity-regulated genes that we tested, but neither of the two neuronal marker genes.

In neuronal cell culture systems, blue light exposure likely induces oxidation due to the presence of compounds such as riboflavin, tryptophan, and HEPES in cell culture media (Spierenburg et al., 1984; Lepe-Zuniga et al., 1987; Edwards et al., 1994; Godley et al., 2005). BrainPhys, the media used in this study, contains both riboflavin and HEPES (Gage and Bardy, 2014; Patent number WO2014172580A1), as does the common neuronal culture medium, Neurobasal Medium (Thermo Fisher Scientific; see manufacturer pamphlet). Therefore, supplementing neuronal culture media with antioxidants (Dixit and Cyr, 2003; Grubb and Burrone, 2010) or altering it to exclude compounds that cause oxidation (Stockley et al., 2017) may mitigate the detrimental effects of blue light in culture systems. Alternatively, sensitive channelrhodopsins (Schoenenberger et al., 2009) can be used to minimize the duration of light exposure and thus its negative effects. Notably, blue light exposure may increase transcription in many as-yet-untested non-neural cultures, as the common cell culture media DMEM also contains riboflavin and HEPES (ThermoFisher). Thus, spurious blue light-induced increases in gene expression may be a concern in any experiment that measures transcription in response to an optogenetic stimulus, including those that use optogenetics to directly increase transcription in non-neural cells (Nihongaki et al., 2015; Polstein and Gersbach, 2015).

The toxic oxidation that occurs in culture media suggests that *in vitro* experiments may be particularly sensitive to blue light exposure. However, oxidation-prone compounds exist within cells and in interstitial fluids, suggesting that light exposure could also affect cells *in vivo*. Consistent with this idea, exposing *C. elegans* to blue light likely produces free radicals within the worm (Bhatla and Horvitz, 2015), and *C. elegans*, planeria, and *D. melanogaster* have free radical-detecting cells that respond to light exposure in the absence of cell culture media (Bhatla and Horvitz, 2015; Guntur et al., 2015; Birkholz and Beane, 2017). Alternatively, it is possible that endogenous opsins or cytochromes, which are expressed in our cultures (Tyssowski et al., 2018) and in the brain (Peirson et al., 2009), play a role in the observed increases in gene expression, in which case we would expect to observe similar increases in activity-regulated gene expression *in vivo*. Indeed, there is some evidence that blue light stimulation in the absence of channelrhodopsin may increase *Fos* expression in the rat brain (Villaruel et al., 2018), although it is not clear whether this is due to light stimulation or other factors, such as the trauma from implanting the optical fiber. Furthermore, blue light exposure changes blood flow in the brain, which may also affect neural gene expression (Rungta et al., 2017). Therefore, it will be important for future work to assess the impact of blue light exposure on neuronal transcription *in vivo*.

## Synthesis

Reviewing Editor: Juan Burrone, King's College London Decisions are customarily a result of the Reviewing Editor and the peer reviewers coming together and discussing their recommendations until a consensus is reached. When revisions are invited, a fact-based synthesis statement explaining their decision and outlining what is needed to prepare a revision will be listed below. The following reviewer(s) agreed to reveal their identity: Matthew Grubb.

This study highlights the importance carrying out adequate controls when performing optogenetic manipulations. Although the findings are potentially important there are a number of issues raised by the reviewers that need to be addressed. Below are the comments by each reviewer.

Reviewer 1

This is potentially important information that the authors are to be commended for taking the time to prepare for publication, instead of just leaving ‘in the drawer’. It seems to present problems primarily for optogenetic experiments that have been poorly designed (i.e. no light-only condition) in the first place, but nevertheless any publication that leads to better-controlled experiments can only be a good thing. However, there are some important issues with the methods, data, analysis and interpretation of the results here that I feel need to be addressed prior to publication.

1) Problems posed by mixed cultures of neurons+glia. All data are based on co-cultures of cortical neurons and glia, but are interpreted as representing neurons only. There are numerous instances throughout the paper where the authors refer to ‘exposing neurons’ or to ‘characterizing the effects of light on transcription in neurons’ and so on, when all manipulations and readouts must have applied to both neurons and glia. The wording therefore needs to be changed to be much more circumspect here. More importantly, though, is the potential for the effects of blue light on gene expression to have been entirely via glial changes. For example, if blue light acted to kill glia, or to reduce overall gene expression in glia, then the effects (normalised to overall Gapdh) would look like a relative increase in expression of ‘neuronal’ genes. In order to state that blue light is affecting gene expression in neurons, the authors need to show that it is not preferentially damaging glia in their cultures, and/or that it does not affect expression of glial-specific genes.

2) Effects on all non-housekeeping genes studied. The authors report changes in just 3 ‘neuronal-activity-dependent’ transcripts, and see blue-light-induced changes in all three, even ‘surprisingly’ in Npas4. Without evidence of at least some (or at least one!) neuron-specific genes whose expression is not altered by blue light stimulation, the data are consistent with interpretations of a non-specific expression increase across all neuronal genes, or - more worryingly - indirect effects via e.g. glial changes (see above).

3) Variability in light intensity. All effects on gene expression are seen with photostimulation with blue light, which is around twice as intense as the two other LEDs used in the study. The lack of effect of green or red light stimulation could be entirely due to diminished light intensity in these channels. I also think the general interpretation that blue=bad and green/red=OK is unfounded from the data presented and potentially damaging - as for any well-designed experiment involving blue light, experimenters using photostimulation with other wavelengths still need to be extremely careful about light-induced non-optogenetic effects and certainly have to include the same light-only stimulation controls in their experiments. Please rewrite so that no-one is tempted to think that all experiments involving green or red illumination are entirely ‘safe’!

4) Use of the term ‘induction’. This is an issue with the title and with the MS in general - the techniques here only allow the authors to detect relative changes in the level of gene expression, and are not sufficient to show that expression is ‘induced’ from zero in any cell.

5) Statistics. Multiple single-sample t-tests are used without any correction for multiple comparisons. E.g. Fig1 involves 6x individual statistical tests that will inflate the false-positive error rate. Please use (e.g. Bonferroni) correction for multiple comparisons, or use tests (e.g. 2-way R-M ANOVA) that allow such comparisons without inflating false positive errors.

6) Neuronal activity. The MEA spike data aren't quantified at all. And they are recorded under very different stimulation conditions to those used for qPCR, with drastically different light exposure times. The methods also lack information about how spikes were recorded and detected. Without well-documented quantitative data comparing spiking across hours-long photostimulation conditions, the authors cannot conclude that their stimuli did not affect neuronal activity.

7) Temperature. The MS should cite the recent EJN article on heat-based photostimulation effects on neurons in acute slices, especially given the much larger changes in temperature seen here (~1deg for 100Hz vs <0.5deg in that study).

8) Details of qPCR methods. Which thermocycler was used, with which cycling protocol? Were technical replicates performed? Were the biological replicates run on the same plate? How was the ‘standard curve’ employed - did the authors use a dilution series of known gene concentration, or was more traditional ddCt used? Finally, how exactly was the ‘fold induction’ calculated?

Reviewer 2

The paper entitled ‘Blue light induces neuronal-activity-regulated gene expression in the absence of optogenetic proteins’ is a good reminder that proper controls for all manipulations need to be performed. This study is therefore important. The authors demonstrate that exposing cultured neurons to large quantities of blue light can alter mRNA levels of select proteins.

Several methodological details are missing which make it difficult to assess whether the effects they report as significant are correctly reported as such, the missing information is specified below.

The somewhat alarmist tone of the paper is a bit exaggerated, it has long been known that blue light affects various cellular and neuronal processes. Blue light in extreme doses should not be applied to tissue, minimizing light dose as much as possible and light-only controls are always needed for optogenetic experiments. The prior knowledge that blue light by itself affects cellular responses has driven the engineering of more sensitive blue-light activated channelrhodopsins such as CheRiff. That being said this is still an interesting and important study but should include at least some references to older similar work.

Specific comments:

1) The broad statement in the introduction p2 last sentence: ‘However, less severe cellular changes that may occur separately from, or in early stages of, cell death have not been characterized’ is simply not true and should be re-phrased. A few examples are Trotter et al., 2017 doi: 10.1039/c6pp00299d who used 5 mW/mm2 for 90s and observed activation of the transcription factor Nrf2 and protection against cytotoxicity; Wade et al., 1988 PNAS 85:9322 used much lower intensity light; a series of papers from the group of Elias Aizenman showed the redox site on the NMDA receptor is responsible for light-induced potentiation of NMDA responses (stronger at lower wavelengths but also above 380nm) and from human studies there are also relevant reports of transcranial light effects, the reference number 27 is also relevant in this regard.

2) More explanation is needed for how ‘fold induction’ is calculated from the already normalized to Gapdh data. Why are not the data normalized to Gapdh simply plotted for each condition (dark, blue light, red light etc.)? These values should be used for the statistical analyses rather than using the ‘log fold change’ (then the statistical comparison can be between groups rather than to ‘log1’).

3) What is the justification for alternating between 2-sided and 1-sided T-tests? Were 1-sided tests used just to be sure the values ‘became significant’?

4) Why do the statistics on log fold change but then plot fold change?

5) What are the n's for the experiments? There are several points on each graph but no explanation if these are from individual wells, or if several wells were pooled from individual plates or several plates pooled on different days.

6) What age (DIV) were the cultures when used?

7) Figure 3A and B states the use of 457 nm light (instead of 475 nm, as in the rest of the manuscript). Is this a typo?

## Author Response

Synthesis Statement for Author (Required):

This study highlights the importance carrying out adequate controls when performing optogenetic manipulations. Although the findings are potentially important there are a number of issues raised by the reviewers that need to be addressed. Below are the comments by each reviewer.

Reviewer 1

This is potentially important information that the authors are to be commended for taking the time to prepare for publication, instead of just leaving ‘in the drawer’. It seems to present problems primarily for optogenetic experiments that have been poorly designed (i.e. no light-only condition) in the first place, but nevertheless any publication that leads to better-controlled experiments can only be a good thing. However, there are some important issues with the methods, data, analysis and interpretation of the results here that I feel need to be addressed prior to publication.

1) Problems posed by mixed cultures of neurons+glia. All data are based on co-cultures of cortical neurons and glia, but are interpreted as representing neurons only. There are numerous instances throughout the paper where the authors refer to ‘exposing neurons’ or to ‘characterizing the effects of light on transcription in neurons’ and so on, when all manipulations and readouts must have applied to both neurons and glia. The wording therefore needs to be changed to be much more circumspect here.

We agree with this criticism and have changed the wording throughout (e.g., to say that we exposed cultures to blue light instead of neurons).

More importantly, though, is the potential for the effects of blue light on gene expression to have been entirely via glial changes. For example, if blue light acted to kill glia, or to reduce overall gene expression in glia, then the effects (normalised to overall Gapdh) would look like a relative increase in expression of ‘neuronal’ genes. In order to state that blue light is affecting gene expression in neurons, the authors need to show that it is not preferentially damaging glia in their cultures, and/or that it does not affect expression of glial-specific genes.

We performed additional experiments to test the effects of light stimulation on astrocyte and microglia marker genes (see Figure 5). We found that 100Hz blue light at high power decreased both astrocyte and microglia marker gene expression and at low power decreased microglia (but not astrocyte) marker gene expression, consistent with glial cell death. We observed a non-significant (p>0.05, q>0.15) but >1.5-change in expression of marker genes in other light stimulation conditions as well.

We do not think that this decrease in glial gene expression results in substantially lower Gapdh expression that could cause a relative (rather than absolute) increase in the expression of neuronal genes. We have several reasons for this reasoning:

(1) Fold change in Gapdh expression is similar between conditions and does not track with the observed patterns of Fos expression (i.e., Gapdh expression does not uniformly decrease in conditions where Fos expression increases). Indeed, the average fold change in Gapdh expression is not more than a 1.5-fold increase or decrease in all conditions.

Figure 1: Fold change in Gapdh for all conditions. Points represent individual biological replicates (n=3-6, from separate dissections) and black lines represent the mean. P-values are from a student's t-test on log fold change values testing the difference from a fold change of 1. q-values are FDR-adjusted p-values. If the observed change in ARG expression were due to a decrease in Gapdh expression, we would expect to see the fold changes for blue light in this plot to be below 1.

(2) We find in new experiments that that expression of neuronal marker genes Thy1 and Tubb3 do not increase in response to light stimulation. If the increases in Fos that we observe were due to light specifically killing glia but not neurons, we would expect to also see light-induced increases in neuronal marker gene expression.

2) Effects on all non-housekeeping genes studied. The authors report changes in just 3 ‘neuronal-activity-dependent’ transcripts, and see blue-light-induced changes in all three, even ‘surprisingly’ in Npas4. Without evidence of at least some (or at least one!) neuron-specific genes whose expression is not altered by blue light stimulation, the data are consistent with interpretations of a non-specific expression increase across all neuronal genes, or - more worryingly - indirect effects via e.g. glial changes (see above).

We have now tested the effect of light exposure on the neuronal marker genes Thy1 and Tubb3 (Figure 4) and we find that their expression does not increase with light exposure, consistent with the possibility that the effect of light is specific to neuronal-activity-regulated genes (and inconsistent with the conclusion that the observed increase in Fos expression is due to death of glia - see above). We also note that we observe a non-significant (p>0.05, q>0.15) decrease in Tubb3, which we think is likely due to the fact that Tubb3 is lowly expressed in some glia.

3) Variability in light intensity. All effects on gene expression are seen with photostimulation with blue light, which is around twice as intense as the two other LEDs used in the study. The lack of effect of green or red light stimulation could be entirely due to diminished light intensity in these channels. I also think the general interpretation that blue=bad and green/red=OK is unfounded from the data presented and potentially damaging - as for any well-designed experiment involving blue light, experimenters using photostimulation with other wavelengths still need to be extremely careful about light-induced non-optogenetic effects and certainly have to include the same light-only stimulation controls in their experiments. Please rewrite so that no-one is tempted to think that all experiments involving green or red illumination are entirely ‘safe’!

We have performed additional experiments using blue- and red-light stimulation at similar intensities (2.2 mW/mm2 red light, as before, and 1.95 mW/mm2 blue light). We find that Fos expression is similar between 1.95 mW/mm2 blue light and 3.9 mW/mm2 blue light (the intensity used in the initial submission), suggesting that blue, but not red, light increases Fos expression, even when controlling for power (Figure 2).

That said, we whole-heartedly agree with the reviewer that it is important to carefully perform the proper controls for any light stimulation, regardless of wavelength. Indeed, we find that red light stimulation may affect glial gene expression (Figure 5). We have altered the text to reflect this view.

4) Use of the term ‘induction’. This is an issue with the title and with the MS in general - the techniques here only allow the authors to detect relative changes in the level of gene expression, and are not sufficient to show that expression is ‘induced’ from zero in any cell.

We have altered the text accordingly.

5) Statistics. Multiple single-sample t-tests are used without any correction for multiple comparisons. E.g. Fig1 involves 6x individual statistical tests that will inflate the false-positive error rate. Please use (e.g. Bonferroni) correction for multiple comparisons, or use tests (e.g. 2-way R-M ANOVA) that allow such comparisons without inflating false positive errors.

We have performed multiple hypothesis adjustment (using FDR) on the list of p-values testing the hypothesis “light stimulation changes gene expression” to account for testing different wavelengths, stimulation frequencies, and genes (i.e., on all p-values in figures 1, 2, 4 and 5). We find that for blue light stimulation, all FDR<0.15, whereas for red/green light stimulation all FDR>0.15. We have reported FDR-derived q-values along with p-values. We chose to use FDR rather than Bonferroni because the Bonferroni correction dramatically increases the rate of false negatives. In this case, we think that a false negative is more detrimental than a false positive, as a false negative could lead researchers to think a certain light treatment is safe, whereas a false positive, in the worst case, will just encourage unnecessary controls.

Furthermore, we would like to point out that the finding under multiple contexts (e.g., time points) that blue, but not red or green, light increases activity-regulated gene expression strengthens our confidence in our conclusion that blue light increases gene expression. This strengthening is not accounted for by multiple hypothesis correction. Therefore, we randomized the data from all experiments testing ARG expression (i.e., we assigned each fold change value to a random light stimulation condition) and determined the proportion of times that we observed p < 0.05 for all blue-light-treated samples but none of the red- and green-light-treated samples, as observed in our actual experiments. In 10,000 repetitions, we never observed that all blue-light-treated samples show increased ARG expression (p<0.0001), indicating that the results we observe are unlikely to have occurred by chance. We have added a description of this analysis to the methods.

6) Neuronal activity. The MEA spike data aren't quantified at all. And they are recorded under very different stimulation conditions to those used for qPCR, with drastically different light exposure times. The methods also lack information about how spikes were recorded and detected. Without well-documented quantitative data comparing spiking across hours-long photostimulation conditions, the authors cannot conclude that their stimuli did not affect neuronal activity.

We agree with the reviewer and have modified the text to include the sentence: “We cannot, however, rule out the possibility that longer exposure to blue light stimulation may increase neuronal activity.’ We have also update the methods to include more information about how spikes were recorded and detected. While we agree that the MEA data are presented in a qualitative way, we think that readers who use optogenetic techniaues will recognize in this qualitative data the absence of the kind of dramatic responses that are typically seen in the presence of an optogenetic actuator.

7) Temperature. The MS should cite the recent EJN article on heat-based photostimulation effects on neurons in acute slices, especially given the much larger changes in temperature seen here (~1deg for 100Hz vs <0.5deg in that study).

Thank you for pointing out this reference. We have included it. We have also included more experiments measuring temperature and have modified our interpretation accordingly (Figure 3B). Specifically, we find that 100Hz red light exposure also raises the temperature of the media but does not alter gene expression.

8) Details of qPCR methods. Which thermocycler was used, with which cycling protocol? Were technical replicates performed? Were the biological replicates run on the same plate? How was the ‘standard curve’ employed - did the authors use a dilution series of known gene concentration, or was more traditional ddCt used? Finally, how exactly was the ‘fold induction’ calculated?

We have added this information to the methods. We would also like to clarify here that we did not use ddCt, but rather made a standard curve for each primer set using a serial dilution of cDNA (of unknown concentration), which allowed us to find the relative change in nucleotide concentration in each cycle (rather than just assuming a doubling as in ddCt) and thus the relative cDNA abundance for each sample.

Reviewer 2

The paper entitled ‘Blue light induces neuronal-activity-regulated gene expression in the absence of optogenetic proteins’ is a good reminder that proper controls for all manipulations need to be performed. This study is therefore important. The authors demonstrate that exposing cultured neurons to large quantities of blue light can alter mRNA levels of select proteins.

Several methodological details are missing which make it difficult to assess whether the effects they report as significant are correctly reported as such, the missing information is specified below.

The somewhat alarmist tone of the paper is a bit exaggerated, it has long been known that blue light affects various cellular and neuronal processes. Blue light in extreme doses should not be applied to tissue, minimizing light dose as much as possible and light-only controls are always needed for optogenetic experiments. The prior knowledge that blue light by itself affects cellular responses has driven the engineering of more sensitive blue-light activated channelrhodopsins such as CheRiff. That being said this is still an interesting and important study but should include at least some references to older similar work.

Specific comments:

1) The broad statement in the introduction p2 last sentence: ‘However, less severe cellular changes that may occur separately from, or in early stages of, cell death have not been characterized’ is simply not true and should be re-phrased. A few examples are Trotter et al., 2017 doi: 10.1039/c6pp00299d who used 5 mW/mm2 for 90s and observed activation of the transcription factor Nrf2 and protection against cytotoxicity; Wade et al., 1988 PNAS 85:9322 used much lower intensity light; a series of papers from the group of Elias Aizenman showed the redox site on the NMDA receptor is responsible for light-induced potentiation of NMDA responses (stronger at lower wavelengths but also above 380nm) and from human studies there are also relevant reports of transcranial light effects, the reference number 27 is also relevant in this regard.

Thank you for pointing out these references. We have included them, and we have removed the inaccurate sentence.

2) More explanation is needed for how ‘fold induction’ is calculated from the already normalized to Gapdh data. Why are not the data normalized to Gapdh simply plotted for each condition (dark, blue light, red light etc.)? These values should be used for the statistical analyses rather than using the ‘log fold change’ (then the statistical comparison can be between groups rather than to ‘log1’).

We have included an explanation for how we calculated fold change in the methods. Fold change is the Gapdh-normalized expression in cultures exposed to light divided by that in unstimulated cultures. We chose to use fold change in our statistical comparisons because while Gapdh-normalized values differ somewhat between biological replicate (perhaps due to dissection-based differences in culture composition), fold changes are quite similar between replicates (this is also true for activity-regulated gene expression activated by membrane depolarization). In this study, we are interested in whether the change in mRNA expression is consistent between replicates, so we have performed statistics on log fold change.

3) What is the justification for alternating between 2-sided and 1-sided T-tests? Were 1-sided tests used just to be sure the values ‘became significant’?

We performed 1-sided tests on all hypotheses testing whether activity-regulated gene expression increased with light stimulation because we wished to specifically test increases. We used 2-sided tests for all other comparisons.

4) Why do the statistics on log fold change but then plot fold change?

We have changed the axes of the graphs to be in log scale. We did statistics on log fold change because fold change is log-normally distributed, so log fold change better fits the assumptions of the t-test.

5) What are the n's for the experiments? There are several points on each graph but no explanation if these are from individual wells, or if several wells were pooled from individual plates or several plates pooled on different days.

The n's are at least 3 biological replicates for each gene expression experiment. The biological replicates are each dissections from separate litters done on separate days. In each replicate, we did 2-4 technical replicates (separate wells on the same plate). In some experiments, we pooled individual wells at the step of mRNA collection, and in others we averaged the values of the technical replicates following qPCR. We have added this information to the methods and updated our figure legends to clarify what we mean by biological replicates.

6) What age (DIV) were the cultures when used?

We used cultures from DIV10-14. This has been added to the methods.

7) Figure 3A and B states the use of 457 nm light (instead of 475 nm, as in the rest of the manuscript). Is this a typo?

Yes-this is a typo. Thank you for pointing it out. We have fixed it.
